# Accelerating health equity: the key role of universal health coverage in the Sustainable Development Goals

**DOI:** 10.1186/s12916-015-0342-3

**Published:** 2015-04-29

**Authors:** Viroj Tangcharoensathien, Anne Mills, Toomas Palu

**Affiliations:** International Health Policy Program, Ministry of Public Health, Tivanond Road, Nonthaburi, 11000 Thailand; London School of Hygiene and Tropical Medicines, Keppel Street, London, WC1E7HT UK; World Bank, East Asia and Pacific Office, Rama 4, Bangkok, 10330 Thailand

**Keywords:** Sustainable development goals, Universal health coverage, Health equity, Health systems strengthening, Health workforce

## Abstract

The Sustainable Development Goals (SDGs), to be committed to by Heads of State at the upcoming 2015 United Nations General Assembly, have set much higher and more ambitious health-related goals and targets than did the Millennium Development Goals (MDGs). The main challenge among MDG off-track countries is the failure to provide and sustain financial access to quality services by communities, especially the poor. Universal health coverage (UHC), one of the SDG health targets indispensable to achieving an improved level and distribution of health, requires a significant increase in government investment in strengthening primary healthcare - the close-to-client service which can result in equitable access. Given the trend of increased fiscal capacity in most developing countries, aiming at long-term progress toward UHC is feasible, if there is political commitment and if focused, effective policies are in place. Trends in high income countries, including an aging population which increases demand for health workers, continue to trigger international migration of health personnel from low and middle income countries. The inspirational SDGs must be matched with redoubled government efforts to strengthen health delivery systems, produce and retain more and relevant health workers, and progressively realize UHC.

## Background

The recognition that health is a precondition for, an outcome of, and an indicator of all three dimensions of sustainable development [1] has led to a series of extensive negotiations among United Nations (UN) member states on the text of the post-2015 Sustainable Development Goals (SDGs; see Box 1). The SDGs follow, and expand upon, the Millennium Development Goals (MDGs), which are due to expire at the end of 2015, though all health-related MDGs continue to be included in the SDGs with newer targets. The SDGs are due to be finalized in September 2015, and will be the result of the largest consultation process by the UN.

Despite the critique on the number of SDGs: 17 goals and 169 targets, all are interlinked, reflecting the fact that sustainable development in a country requires multidimensional and multisectoral policy interventions. These include addressing poverty, hunger, food insecurity and malnutrition, environmental protection, quality education, universal health coverage (UHC), employment, and decent work. All of these issues are embraced within an equity framework and interwoven with health considerations.

Take the case of malnutrition. Children with severe malnutrition have a higher mortality risk; malnutrition accounts for 45% of total annual child mortality [2]. While management of acute malnutrition within the health sector is cost-effective [3], food and nutritional security realized by sustainable resilient agriculture and improved capacity to adapt to climate change, drought, flooding, and disasters in SDG2, is equally important and synergistic. Or consider the case of tobacco as a significant contributor to the non-communicable disease (NCD) epidemic. Strengthening implementation of the Framework Convention on Tobacco Control and controlling harmful use of alcohol will face industry resistance, and in some countries is hampered by free trade agreements and trade interests dominating health goals. Addressing these cross-sectoral complexities requires strong leadership, active civil society organizations, and effective intersectoral actions to ensure that a health lens is taken by other policies.

The 13 targets (nine specific and four cross-cutting) of the health goal in SDG3 are raised to a level much higher than in the MDGs, such as reducing the maternal mortality rate to no more than 70 per 100,000 live births, ending preventable deaths in newborns and children, reducing one-third of premature mortality from NCDs, halving global deaths and injuries from road traffic accidences, and achieving UHC.

UHC is a significant SDG health target combining financial protection against catastrophic health spending and medical impoverishment as well as ensuring access to essential services. It is both a measurable goal in itself with significant contribution to welfare valued by societies, as well as an important means for achieving the other SDG3 health targets. It is also high on the global agenda, as reflected in the 2012 United Nations General Assembly Resolution. To reflect this key role of UHC this commentary reviews different trajectories countries have taken in making progress toward UHC, and accelerating the achievement of health equity, financial protection, and long-term sustainability [4].

### Universal health coverage: different trajectories

Although countries take different routes in making progress toward UHC, based on their socio-economic and political context, a common trend emerges: different financing sources are used to cover different population groups. Public and private sector employees are covered by payroll-tax financed contributory schemes, often taking the form of mandatory social health insurance (SHI). The poor are usually covered by tax-financed mechanisms either directly managed by the Ministry of Health or as part of the SHI as in Vietnam and the Philippines. Coverage of the large informal sector is financed by a range of funding sources; from full premium contributions by households, to partial and fully tax subsidized premiums. Most countries in Asia gradually shift from full contributions to tax funding depending on the government fiscal space and, most importantly, political leadership. Countries find it difficult to expand coverage of the informal sector through contributory schemes because of ineffective mechanisms to enforce contribution payment [5].

Another trajectory is in countries where policy choice is to achieve UHC via services that are (in theory) provided free of charge in public health facilities. In this trajectory, in some countries public spending on health may not match the increased demand for health services, resulting in high levels of household out-of-pocket payments, for example 45% of total health spending in Sri Lanka [6]. Also, wealthier members of the population may opt out of government services, preferring to pay out of pocket for private services (Malaysia). But, on the other hand, in the Pacific Island States, publicly provided health services at relatively high cost to the governments have actually minimized out-of-pocket payments by the population.

The design and inter-relationship between health delivery and financing have major ramifications for health systems performance. Evidence from Organization for Economic Cooperation and Development (OECD) countries suggests that public contract where there is a direct relationship between purchaser organization and healthcare providers, or reimbursement systems where the purchaser organization reimburses patients for their medical bills, are more efficient than public integrated systems where healthcare providers are owned by a purchaser organization [7]. But this efficiency is also a function of strong institutions in OECD countries compared to those in developing countries.

### Universal health coverage: contribution to health equity

To achieve a favorable UHC outcome, strengthening physical access by improving geographical coverage of health services, and financial access by extension of financial risk protection mechanisms, are two essential parallel synergistic interventions [8]. The higher the coverage of skilled birth attendance (SBA), the smaller the rich-poor disparities [9]. In countries with very low SBA coverage, that is, less than 30%, the rich-poor disparities are large, at around 60 percentage points. A smaller disparity, less than 20 percentage points, is observed in countries having high coverage.

Where 100% SBA coverage is reached, as in Thailand, there are no gaps whether by maternal education or by socio-economic status [10]. In Thailand, universal coverage of Maternal and Child Health (MCH) services resulted in rapid reduction in the rich-poor gap of child mortality between the 1990 and 2000 censuses [11]. Relative inequalities tend to be larger in countries with lower overall levels of health care use [12]. The US Affordable Care Act coverage expansion has resulted in improved access to a usual care provider for millions of black and Hispanic Americans, and reduced the likelihood of going without care because of cost [13].

Functioning close-to-client primary health care (PHC) which the majority of the poor can access [14] acts as a major hub in translating UHC political intentions into pro-poor outcomes such as service utilization and government subsidies [15]; a comprehensive benefit package results in high levels of financial risk protection, preventing non-poor households from becoming poor due to medical payments [16].

### Health workforce: a backbone of health systems

The health workforce is critical to functioning health services. Shortages and maldistribution of the health workforce, a common problem facing many MDG off-track countries, has been a constant challenge despite the 2008 Kampala commitment [17]. Investment in the health workforce remains low, with large gaps between demand and supply; health workforce planning is often weak without intersectoral coordination; policies on retention of the health workforce in rural areas and within countries are not fully implemented; scaling and transforming health professional education is at an early stage of reform [18].

Future projections demonstrate that low income countries will face a widening gap between the supply and need for health workers, but have limited capacity to employ more workers, even if supply can be increased. Upper middle income countries will face a similar widening gap, but created by demand factors, which could drive up health care costs or encourage in-migration of health workers. Projection by the International Labour Organization (ILO) shows that 10.3 million additional health workers globally are required to close the current gaps and ensure universal health coverage, of which 7.1 million are needed in Asia and 2.8 million in Africa [19]; these gaps are hardly met unless governments have strong commitments to produce and retain health workers in countries. OECD countries are the major destinations for international migration of health workers, often the highly skilled workers from low and middle income countries. Demand for health workers in high income and emerging countries due to aging and needs for long-term care stimulates international migration. This is exacerbated by the unresolved “push factors” in source countries, such as low pay, lack of career paths, and poor working conditions. Despite the World Health Assembly adopting by consensus the WHO Global Code of Practice on International Recruitment of Health Personnel [20], implementation of the Code is suboptimal, as reflected by the first report of the Code’s implementation [21]. But on the other side of equation is the macro-economic calculus of the professional migration from the demographic dividend countries that goes beyond individual push and pull factors. In the Philippines, the remittances from migrants, of which health professionals make up a significant part, contribute more than 10% to the gross national income (GNI). In a global economy, win-win situations may be possible if importing countries adhere to the Code, and donor countries organize their health professional educations system and labor markets so that local populations’ access to qualified health professionals does not suffer.

Skill mix, cadre mix, and task shifting [22], clinical and public health competency, performance, and social accountability are as important as numbers of health workers. These require transformation of the instructional and institutional dimensions of health professional education systems. A more diverse composition of the health workforce, and expansion of health workers in the community and of mid-level health workers, needs careful planning [23].

### Finding fiscal space

Progressively achieving UHC will require a significant increase in public investment. Countries would need to systematically review the opportunities under the five domains of fiscal space creation [24]. Macroeconomic conditions remain challenging over the medium term with slow growth in developed countries and slowing growth in Asia. But Africa has just had a decade of the fastest economic growth that should create opportunities for fiscal space for health. The recent Lancet Commission report on Global Health 2035 makes a strong economic case for health that should facilitate greater prioritization of health by the economic ministries in countries [25]. The Philippines has recently demonstrated success in raising additional resources for health through a sin tax reform for tobacco and alcohol, 80% of the revenues accruing to speeding up progress toward the UHC. In spite of global economic problems, the UK has just reaffirmed its commitment to allocate 0.7% of the gross domestic product (GDP) to overseas development assistance [26], and the recent Chatham House Global Health Financing report [27] calls for 0.15% to go toward health. But perhaps the most untapped resource for increasing fiscal space for health is efficiency gains from existing allocations by using evidence-based approaches to priority setting, resource allocation, performance-oriented provider payment mechanisms, and strengthened public financial management and accountability.

## Conclusion

UHC and the health workforce are two among 13 health targets in the SDGs, and jointly contribute to the achievement of the SDGs. The upcoming health targets in the SDGs, more inspirational and demanding than the previous health-related MDGs, are achievable only when countries demonstrate investment in health systems strengthening beyond the rhetorical statements made at the United Nations General Assembly by Heads of State.

## Box 1: The Proposed Sustainable Development Goals

People are at the center of sustainable development. The promise is to strive for a world that is just, equitable, and inclusive where all stakeholders have to work together to promote sustained and inclusive economic growth, social development, and environmental protection benefiting all without distinction of age, sex, disability, culture, race, ethnicity, origin, migratory status, religion, economic, or other status [28].

Based on these inspirations, the 17 interconnected sustainable development goals are proposed. These will be finalized in September 2015.

**GOAL 1** End poverty in all its forms everywhere

**GOAL 2** End hunger, achieve food security and improved nutrition, and promote sustainable agriculture

**GOAL 3** Ensure healthy lives and promote well-being for all at all ages

**GOAL 4** Ensure inclusive and equitable quality education and promote lifelong learning opportunities for all

**GOAL 5** Achieve gender equality and empower all women and girls

**GOAL 6** Ensure availability and sustainable management of water and sanitation for all

**GOAL 7** Ensure access to affordable, reliable, sustainable, and modern energy for all

**GOAL 8** Promote sustained, inclusive, and sustainable economic growth, full and productive employment, and decent work for all

**GOAL 9** Build resilient infrastructure, promote inclusive and sustainable industrialization, and foster innovation

**GOAL 10** Reduce inequality within and among countries

**GOAL 11** Make cities and human settlements inclusive, safe, resilient, and sustainable

**GOAL 12** Ensure sustainable consumption and production patterns

**GOAL 13** Take urgent action to combat climate change and its impacts

**GOAL 14** Conserve and sustainably use the oceans, seas, and marine resources for sustainable development

**GOAL 15** Protect, restore, and promote sustainable use of terrestrial ecosystems, sustainably manage forests, combat desertification, and halt and reverse land degradation and halt biodiversity loss

**GOAL 16** Promote peaceful and inclusive societies for sustainable development, provide access to justice for all, and build effective, accountable, and inclusive institutions at all levels

**GOAL 17** Strengthen the means of implementation and revitalize the global partnership for sustainable development

## Abbreviations

GDP: gross domestic product
GNI: gross national income
ILO: International Labour Organization
NCD: non-communicable disease
OECD: Organization for Economic Cooperation and Development
PHC: primary health care
SBA: skilled birth attendance
SDG: Sustainable Development Goal
SHI: social health insurance
UHC: universal health coverage
